# Chronic exposure to perfluorohexane sulfonate leads to a reproduction deficit by suppressing hypothalamic kisspeptin expression in mice

**DOI:** 10.1186/s13048-021-00903-z

**Published:** 2021-10-27

**Authors:** Xiaorui Yin, Tingting Di, Xinyuan Cao, Zhengnan Liu, Jingyan Xie, Suyun Zhang

**Affiliations:** 1 Department of Obstetrics and Gynecology, Nanjing First Hospital, Nanjing Medical University, Changle Road 68, Nanjing, 210006 China; 2 Department of Pharmacology, Institute of Translational Medicine, School of Medicine, Yangzhou University, Yangzhou, 225001 China; 3 Experimental Teaching Center of Basic Medicine, Nanjing Medical University, Nanjing, 210006 China

**Keywords:** Perfluorohexane sulfonate, Kisspeptin, Reproduction, Hypothalamus

## Abstract

**Background:**

Perfluorohexane sulfonate (PFHxS) is a six-carbon perfluoroalkyl sulfonic acid found as an environmental contaminant. This study aims to investigate the effects of PFHxS exposure on female reproduction and the underlying mechanism in mice.

**Methods:**

Eight-week-old ICR mice were divided randomly into four groups administered corn oil (vehicle) and PFHxS at doses of 0.5, 5, and 50 mg/kg/day for 42 days by intragastric administration. Body weight, ovarian weight, estrous cycle, follicle counts, and serum sex hormone levels were evaluated. The expression of kisspeptin and gonadotropin releasing hormone (GnRH) in the hypothalamus was also detected.

**Results:**

Compared to vehicle exposure, 5 mg/kg/day PFHxS treatment prolonged the estrous cycle, especially the duration of diestrus, after 42 days of treatment. The numbers of secondary follicles, antral follicles and corpus lutea were significantly reduced in the PFHxS-treated mice. Moreover, compared with the control mice, the PFHxS-treated mice showed decreases in the serum levels of follicle-stimulating hormone (FSH), luteinizing hormone (LH), and estrogen (E2), and reduced GnRH mRNA levels, along with the lack of an LH surge. Furthermore, the PFHxS-treated mice had lower levels of kisspeptin immunoreactivity and kiss-1 mRNA in the arcuate nucleus (ARC) and anteroventral periventricular nucleus (AVPV) than the control mice. After intraventricular administration of kisspeptin-10, the numbers of secondary follicles, antral follicles and corpus lutea recovered, along with the levels of GnRH mRNA, FSH, and LH in the mice treated with 5 mg/kg/day PFHxS.

**Conclusion:**

These results indicate that chronic exposure of mice to 5 mg/kg/day PFHxS affects reproductive functions by inhibiting kisspeptin expression in the ARC and AVPV regions, leading to deficits in follicular development and ovulation.

**Supplementary Information:**

The online version contains supplementary material available at 10.1186/s13048-021-00903-z.

## Background

Perfluorohexane sulfonate (PFHxS) belongs to the family of perfluoroalkyl compounds (PFASs) that are widely used in the production of various industrial and chemical products, including paper, upholstery, food packaging, and firefighting foam [1]. Due to their global distribution, high persistence, bioaccumulation potential, and strong toxicity, these compounds have been recommended to be listed as persistent organic pollutants by the European Chemicals Agency in accordance with the Stockholm Convention [2]. Abundant research from various laboratories has shown that multiple adverse effects, such as tumour induction, fetal growth interruption, neurotoxicity, and endocrine disruption are related to the contact with PFASs [3]. PFHxS has been detected in soil, drinking water, several consumer products, and animals [4]. Human exposure to PFHxS is mainly occurs through ingestion of contaminated water and food. This chemical can also be passed through the placenta to the embryo and through breast milk to infants [5]. PFHxS is persistent and poorly metabolized with estimated half-lives of 1 month, 4 months, and 7.3 years in mice, monkeys, and humans, respectively [6, 7].

The concentration of PFASs in circulating blood is similar to those in ovarian follicular fluid. This finding indicates that serum samples can be used as an appropriate substitute index for the ovarian exposure level [8]. The level of PFHxS was negatively correlated with the concentration of triiodothyronine (T3) and thyroxine (T4) in 202 serum samples from Chinese individuals [9]. A previous study with human breast cancer MCF-7 cells showed that PFHxS is a weak agonist of estrogen receptors (ESR1) and a potential weak antagonist of the androgen receptor (AR) [10]. Another study showed mild to moderate liver and thyroid hypertrophy with PFHxS exposure at two different doses (3 and 10 mg/kg) [11]. Although several studies have evaluated the effects of PFHxS on reproduction, there are very few reports exploring the mechanism of the imparted toxicity.

The hypothalamic-pituitary-gonadal (HPG) axis regulates ovarian activity during the menstruation period. Gonadotropin releasing hormone (GnRH) regulates the release of follicle-stimulating hormone (FSH) and luteinizing hormone (LH), and these sex hormones, in turn, regulate FSH, LH and GnRH. Kisspeptin, also known as metastin, serves as a gatekeeper of puberty and is mainly expressed in the hypothalamus of rodents, especially the arcuate nucleus (ARC) and the anterioventral periventricular nucleus (AVPV) areas [12], in addition to the paraventricular nucleus and amygdala [13]. Kisspeptin is a 145 amino acid neuropeptide encoded by the kiss-1 gene, and is cleaved into short peptides of different lengths to function. Approximately 90% of GnRH neurons express the kisspeptin receptor, also known as G protein-coupled receptor 54 (GPR54) [14], which can be strongly activated by kisspeptin neurons in the AVPV [15]. GPR54 is mainly expressed in rodent brains [16], especially GnRH neurons [17], along with the liver and pancreas [18]. Kisspeptin is also involved in the feedback regulation of GnRH/LH by estrogen (E2) [19]. Kisspeptin can regulate the secretion of GnRH through the activation of GPR54 in the hypothalamus and then participates in reproductive endocrine functions [20]. Given the effect of kisspeptin on reproductive function, we hypothesized that the toxicity of PFHxS may be related to the expression of kisspeptin.

Among several PFASs currently in use, only perfluorooctane sulfonic acid (PFOS) is regulated under the Stockholm Convention. There are very few reports related to the uptake and elimination of PFHxS, and its effects on reproductive and endocrine functions remain unknow [21]. In the present study, we tried to establish a mouse model of chronic exposure to PFHxS. The effects of PFHxS on the general state of mice, follicular development, normal ovulation, HPG axis regulation, and its possible molecular mechanism were explored.

## Methods

### Animals

We complied with the approval of Nanjing Medical University and Institutional Animal Care and Use Committee (IACUC) for the utilization of animals (ethics approval number: DWSY-2000528). Our research used eight-week-old female ICR (defined as Institute of Cancer Research) mice, which were purchased from Oriental Bio Service, Nanjing, China. These mice were selected based on another perfluorooctanate (PFOA) model in our department [22]. All mice were housed in the Laboratory Animal Research of Nanjing Medical University, where all the instruments and food were supplied to raise the mice. To minimize additional exposure to endocrine disrupting chemicals, we adopted cages of stainless steel and wooden plates to house the mice. The temperature was 23 ± 2 °C and the humidity was 55 ± 5% along with a light/dark period of 12:12 h. Food and water were freely available without interruption. Fed as dry pellets to limit spillage, diets, which were packed in vacuum, were kept in a room at 8 °C before use. Mice were allowed to acclimatize for 1 week before the start of the study. Mouse body weights were measured daily. The estrous cycle was monitored every day at 0800–0900 h and assessed by vaginal cytology according to a previously reviewed procedure [23]. All mice were sacrificed by cervical decapitation under anaesthesia with ketamine (80 mg/kg, i.p.).

A total of 84 mice were used in our study, which were all identified by earmark numbers. The mice were divided into three experimental groups: The first group (6 control mice and 18 PFHxS-treated mice) was used to examine whether the exposure to PFHxS alters overall size, hair condition, body weight, ovarian weight, estrous cycle and ovarian morphology. The second group (24 control mice and 24 PFHxS-treated mice) was used to investigate whether the exposure to PFHxS at dose of 5 mg/kg/d affects the function of HPG axis, as well as the kisspeptin expression in the hypothalamus. The third group (12 PFHxS-treated mice) was used to evaluate the involvement of Kp-10 in PFHxS-altered ovarian morphology and the function of HPG axis.

### Administration of drugs

We purchased PFHxS (#50929) and kisspeptin-10 (#M2816) with the purity higher than 95% from Sigma-Aldrich (Copenhagen, Denmark). After dissolving in dimethyl sulfoxide (DMSO), PFHxS was diluted using corn oil (eventual concentration of 0.5% DMSO). Control mice were given corn oil containing 0.5% DMSO, and the mice in the three groups that were treated with PFHxS were given corn oil with 0.5, 5, and 50 mg/kg/d PFHxS respectively for 42 days. PFHxS was administered to the mice by oral gavage.

For repeated intracerebroventricular (i.c.v.) injection of kisspeptin-10 (Kp-10), the mice were anaesthetized with ketamine (80 mg/kg, i.p.) and then placed into a stereotaxic instrument (Stoelting, Wood Dale, IL, USA). A small hole (2 mm diameter) was drilled in the skull using a dental drill. A guide cannula (26-gauge, Plastics One, Roanoke, VA, USA) was implanted into the right lateral ventricle (0.3 mm posterior, 1.0 mm lateral and 2.5 mm ventral to Bregma) and anchored to the skull with three stainless steel screws and dental cement. On day 3 after surgery, the dummy cannula was removed from the guide cannula, and replaced by infusion cannulas (30 gauge) connected by polyethylene tubing (PE10; Becton Dickinson, Sparks, MD, USA) with a stepper-motorized microsyringe (Stoelting, Wood Dale, IL, USA). The Kp-10 (1 nmol/3 μl) or 0.9% saline was injected daily for 7 successive days [24].

### Ovarian morphology

With normal histological protocols, we fixed ovaries in paraformaldehyde at a concentration of 4% for 24 h. Paraffin-embedded ovaries were sliced at a thickness of 5 μm. After dyeing the slices with hematoxylin & eosin (HE), we placed them on slides. The classification of follicular stages was made following the morphological criteria as described previously [25]. The numbers of follicles (primordial, primary, secondary, antral follicles) and corpora lutea were counted in every 5th section (25 mm apart) and then multiplied by 5 to obtain the total number in each ovary [25]. Only follicles containing an oocyte with a visible nucleus were counted to avoid double counting.

### Measurements of serum hormones

Orbital blood (200 μl) was collected under anaesthesia of ketamine (80 mg/kg, i.p.) on the day of diestrus, kept at room temperature for 30 min, centrifuged (1500 g) at a temperature of 4 °C for 10 min, and used for analysis. To explore the LH-surge, we acquired orbital blood (100 μl each time) was acquired at 1600, 1700, and 1800 h of proestrus. Serum concentrations of FSH, LH, E2 and T4 were surveyed by commercial enzyme-linked immunosorbent assay (ELISA) kits (USCN Life Science, Houston, TX, USA) according to the manufacturer’s protocols. Intra-assay and inter-assay variabilities were both under 15%. The sensitivities were 2.0 pg/mL for E2, 0.2 ng/mL for LH, and 0.4 ng/mL for FSH.

### Immunohistochemistry of kisspeptin neurons

Mice were narcotized by ketamine (80 mg/kg, i.p.) and perfused with precooled phosphate-buffered saline (4 °C) through the left ventricle, and fixed with 4% paraformaldehyde. The entire brain was removed and fixation continued for 24 h. For frozen sections, the brains were transferred gradually into 15 and 30% sucrose. After sinking to the bottom in 30% sucrose, the brains were fixed in optimal cutting temperature (OCT) compound. Coronal sections (30 μm) through the AVPV (from bregma + 0.62 to + 0.02 mm) [26] and ARC (from bregma − 1.22 to − 2.80 mm) [27, 28] were sliced continuously with a cryostat (Leica, Heidelberg, Germany). The brain slices were completely immersed in the antigen retrieval solution and incubated in a constant temperature water bath at 80 °C for 20 min. Subsequently the slices were preincubated in 1% standard foetal goat serum for 1 h, followed by incubation with a rabbit anti-kisspeptin polyclonal antibody (1:1000, Millipore, Billerica, MA, USA) at a temperature of 4 °C for 24 h. The next day, the slices were incubated with biotin-conjugated goat anti-rabbit IgG (1:400; Vector Laboratories, Burlingame, CA, USA) at room temperature for 2 h and then treated with Ni-3, 3′-diaminobenzidine (DAB). Finally, the coloured section were attached to a glass slides, dried, and mounted with a neutral resin, and the kisspeptin-positive (kisspeptin+) neurons were observed by conventional light microscopy (Olympus DP70; Olympus, Tokyo, Japan).

### Reverse transcription quantitative polymerase chain reaction (RT-qPCR)

Before the storage at a temperature of − 80 °C until observation, there was a collection of brain sections in the frozen state at a thickness of 200 μm, which included the preoptic area (POA) region (0.76 mm in front of the bregma as well as 0.50 mm behind the bregma) along with the anteroventral periventricular nucleus (AVPV) region (0.50 mm in front of the bregma as well as 0.02 mm behind the bregma) at proestrus, and the arcuate nucleus (ARC) region (− 1.46 mm in front of the bregma as well as − 1.70 mm behind the bregma) at diestrus. Using Trizol reagent (Invitrogen, Carlsbad, CA, USA), we isolated the overall RNA from areas of the POA, AVPV, and ARC. In the light of the guidance of NovoScript® 1st Strand cDNA Synthesis SuperMix (gDNA Purge) (Novoprotein Scientific, Shanghai, China), we carried out the reverse transcription. The synthesized cDNA was kept at a temperature of − 20 °C before the qPCR. We adopted the following primers as described in a previous study [24]: GnRH F-5′-GGGAAAGAGAAACACTGAACAC-3′, R-5′-GGACAGTACATTCGAAGTGCT-3′; kiss-1 F-5′-GAATGATCTCAATGGCTTCTTGG-3′, R-5′-TTTCCCAGGCATTAACGAGTT-3′; glyceraldehyde 3-phosphate dehydrogenase (GAPDH) F-5′-ACCACAGTCCATGCCATCAC-3′, R-5′-TCCACCACCCTGTTGCTGTA-3′. For every gene and GAPDH (as the internal control), each specimen was analysed in triplicate. By the 2^−ΔΔCT^ approach and normalized expression of GAPDH, we confirmed gene expression relatively.

### Statistical analyses

Using SPSS (IBM, Chicago, IL, USA), a 20.0 version, we analysed the whole data displayed as the mean ± SEM (standard error of the mean). All statistical analyses passed the Shapiro-Wilk normality test and Grubbs’ outlier test (no data points were excluded). Two-group analysis was performed by Student’s t-test (normally distributed data). Sample sizes in all experiments were calculated via an a priori sample size calculation with G*Power for Windows 10 software [29]. The generic binomial test was chosen under the “exact” test family tab to evaluate differences between experimental groups. A priori type of power analysis was selected. An alpha-value of 0.05, power of 0.80, p1 of 0.60, and p2 of 0.98 were used to calculate the sample sizes used throughout experiments. Significant differences within groups were determined by the application of a multiple-comparison one-way or two-way analysis of variance (ANOVA), with Bonferroni corrections. Using Student’s t-test, we made comparisons between the two groups. A value of *P* < 0.05 was considered statistically significant. At the fewest, there were three repetitions for each test.

## Results

### Effect of PFHxS exposure on the general state of mice

**Fig. 1:**
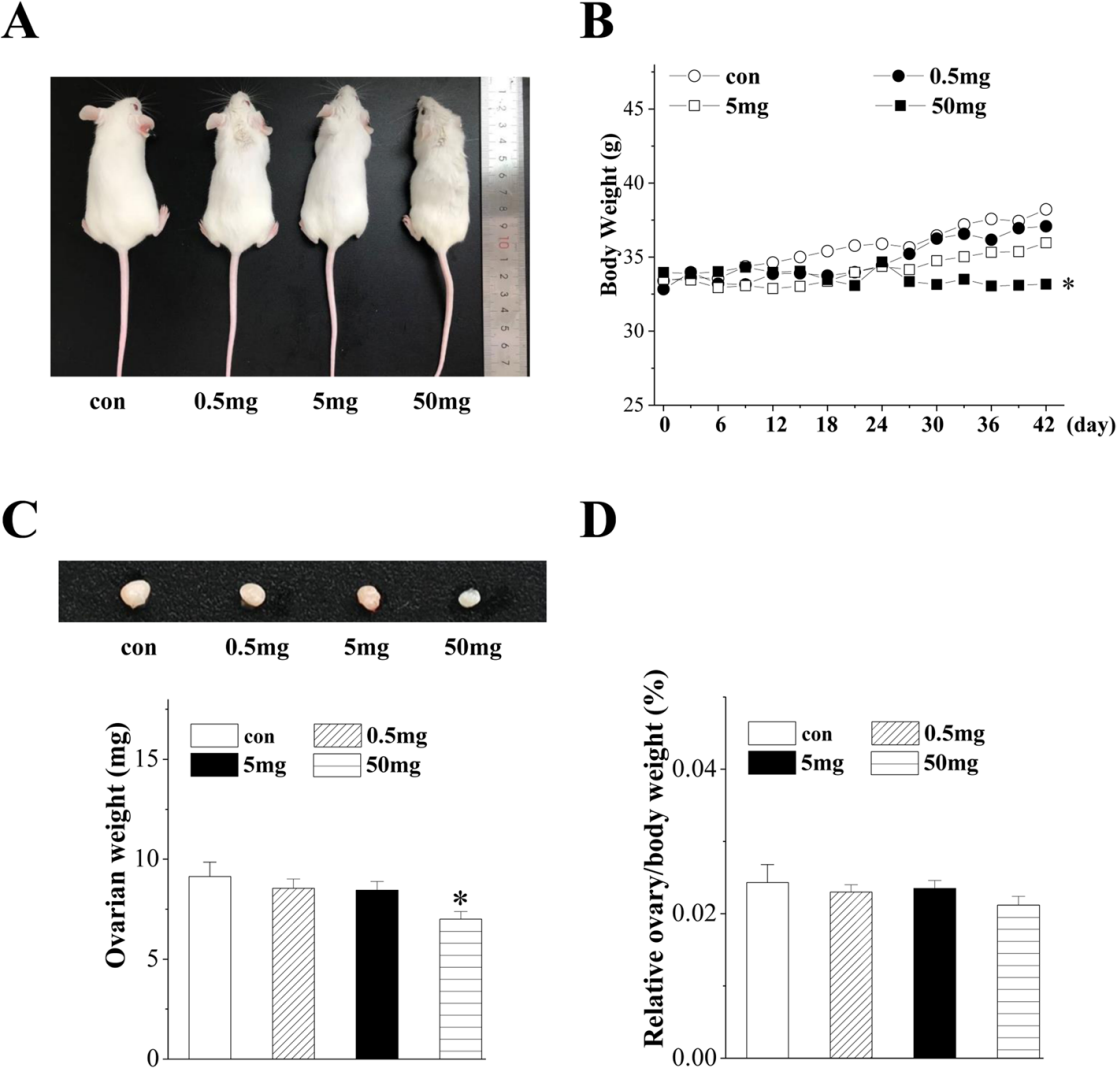
The general state of the mice after chronic exposure to PFHxS. **A** Overall size and hair condition. **B** Body weights (g) of the mice. **C** Ovarian shapes and weights (mg) of mice. **D** Relative ovarian weight to body weight. *P < 0.05 vs. the control mice (*n* = 6/group, one-way ANOVA)

Figure 1 shows the general state, body weights, ovarian weights and ovary to body weight of the control and PFHxS-treated mice. PFHxS exposure at a dose of 50 mg/kg/d changed the general state of mice, such as their overall size and hair condition (Fig. 1A). In addition, the mean body and ovary weights were significantly decreased in the 50 mg/kg/d group (body: *P* = 0.011; ovary: *P* = 0.023; Fig. 1B-C, *n* = 6). The mean body weights of 0.5 and 5 mg/kg/d PFHxS-treated mice were not different from the control mice (*P* > 0.05), nor were the mean ovary weights (*P* > 0.05). There were no statistically significant differences among the four groups in the relative ovarian weight to body weight (F_(3,44)_ = 0.511, *P* > 0.05, *n* = 6; Fig. 1D). To avoid the influence of systemic toxicity on reproductive and endocrine functions, we analysed only mice treated with 0.5 and 5 mg/kg/d PFHxS for subsequent experiments.

### Effect of PFHxS exposure on the estrous cycle

**Fig. 2:**
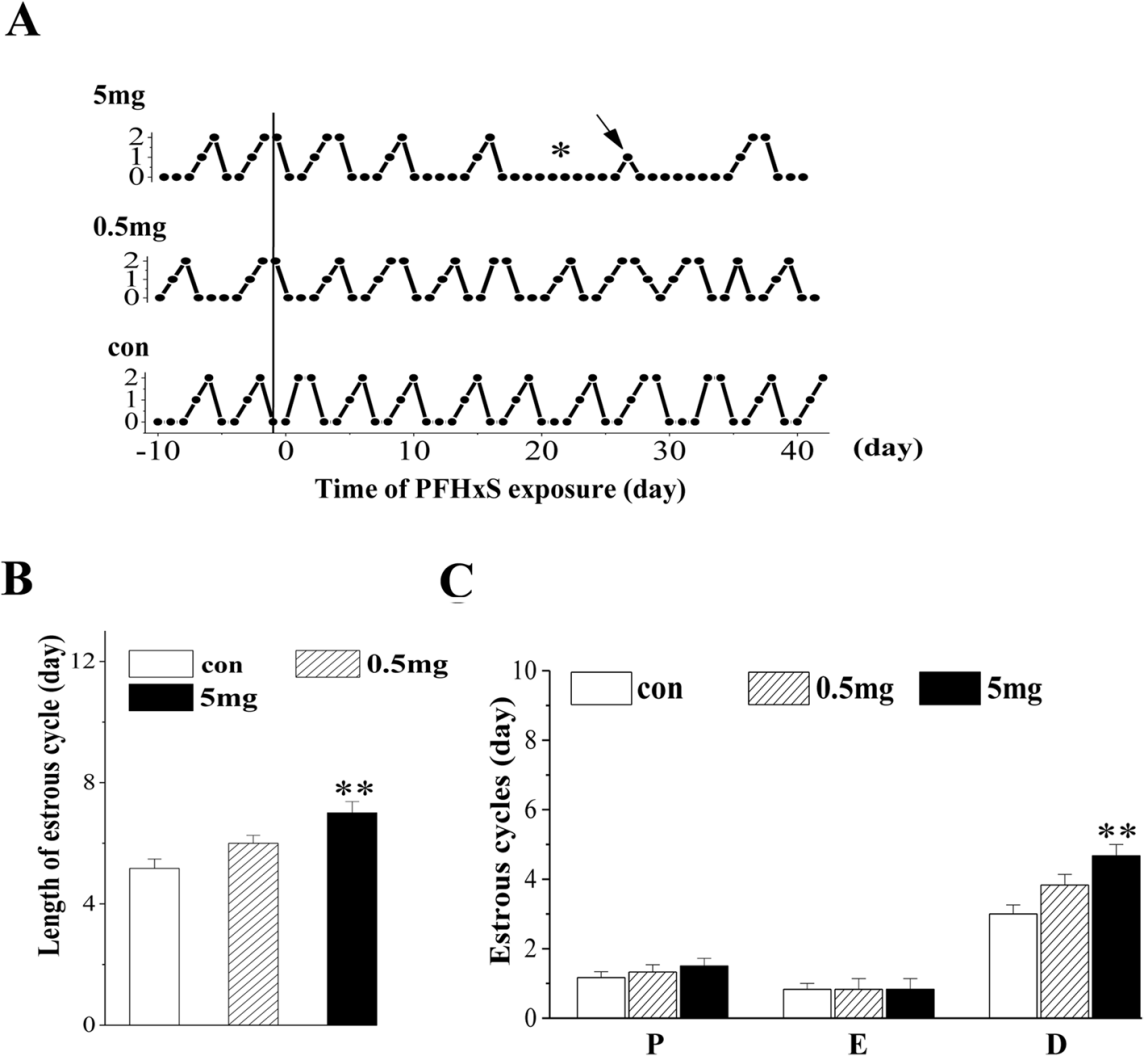
Changes in the estrous cycle after chronic exposure to PFHxS. **A** A representative graph of estrous from each group, 10 days before PFHxS administration and 42 days during the PFHxS treatment. Spots connected separately represent the diestrus time (0), proestrus (1), or estrus (2). * persistent diestrus; ↓ proestrus loss. **B** The average length (day) of a single estrous cycle. **C** The average time (day) of proestrus (P), estrus (E), and diestrus (D) separately, per estrous cycle. **P < 0.01 vs. the control mice (*n* = 6/group, one-way ANOVA)

By vaginal cell observation, we surveyed the cycles of estrous. “Regular cyclers”, which were animals having regular periods lasting 4 to 5 days, had 1 day at proestrus, 1 day at estrus and 2 to 3 days at diestrus (1 day at metestrus) (Fig. 2A). In comparison with the regular estrous cycle observed in the control mice, the PFHxS-treated mice at a dose of 5 mg/kg/d displayed a prolonged estrous cycle (*P* = 0.002, *n* = 6; Fig. 2B), especially an increased duration of diestrus (*P* = 0.003, *n* = 6; Fig. 2C). No significant changes of the estrous cycle were evident in the mice treated with 0.5 mg/kg/d PFHxS (*P* > 0.05; Fig. 2).

### Effect of PFHxS exposure on follicle development and ovulation

**Fig. 3:**
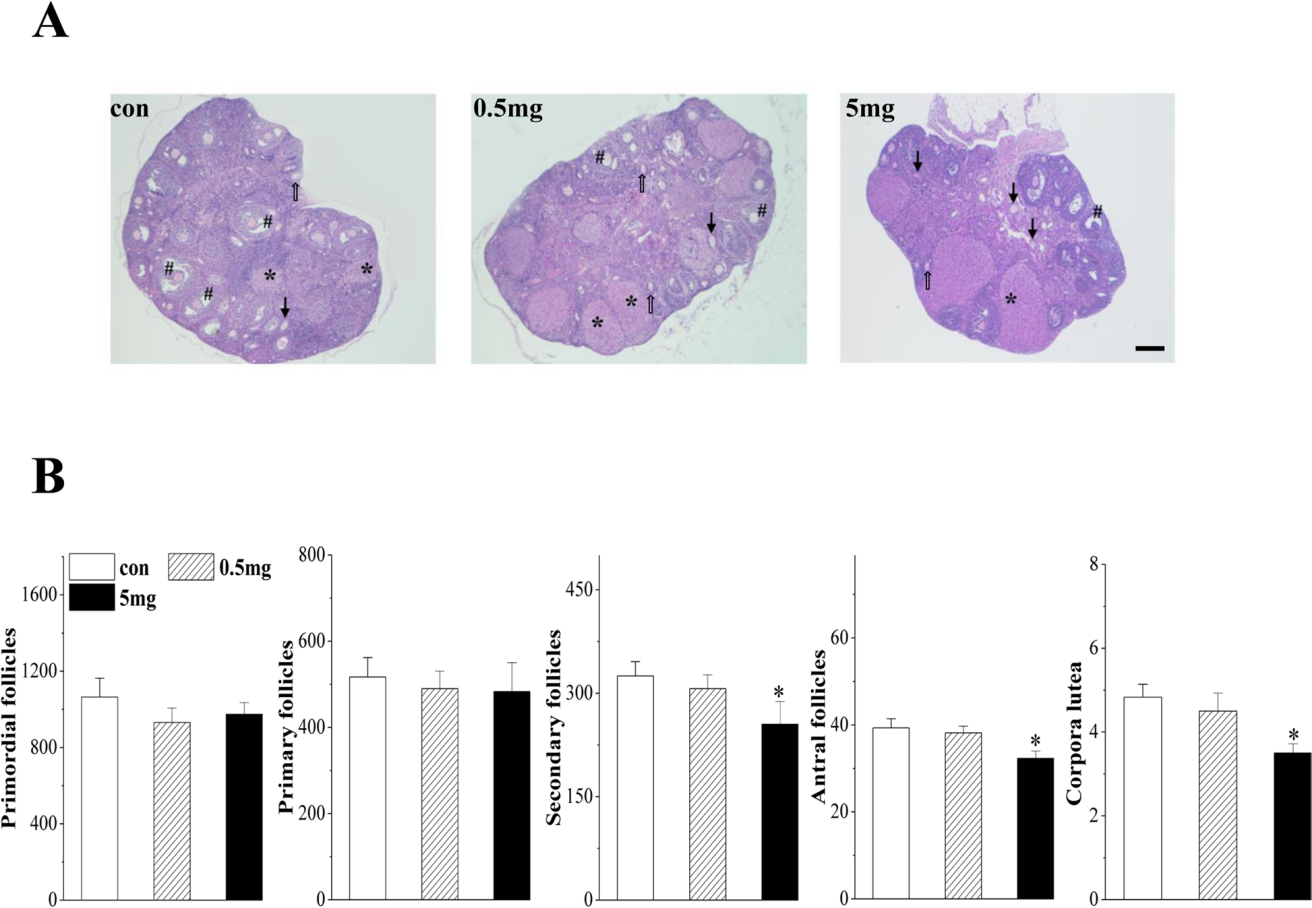
Follicle development and ovulation after chronic exposure to PFHxS. **A** Representative images of ovaries stained with hematoxylin and eosin from the control and PFHxS-treated mice. * for corpora lutea; # for antral follicles; ⇧ for secondary follicles; ↑ for atretic follicles. Scale bars = 200 μm. **B** Average number of primordial follicles, primary follicles, secondary follicles, antral follicles and corpora lutea in each group. *P < 0.05 vs. the control mice (*n* = 6/group, one-way ANOVA)

To examine the influence of chronic exposure to PFHxS on follicle development and maturation, we counted the number of primordial, primary, secondary and antral follicles in addition to the corpora lutea. Samples from the control and 0.5 mg/kg/d PFHxS-treated mice displayed a typical diestrus period (Fig. 3A). We observed extensive corpora lutea in these two groups, proving that there was recent obvious ovulation. The mice treated with 5 mg/kg/d PFHxS displayed a remarkable decrease in the number of secondary follicles (*P* = 0.040, *n* = 6), antral follicles (*P* = 0.025, *n* = 6) and corpora lutea (*P* = 0.022, *n* = 6), without a significant difference in primordial (*P* > 0.05) and primary (*P* > 0.05) follicles (Fig. 3B). Because there was no obvious adverse effect on follicular development and maturation in the 0.5 mg/kg/d group, the mice treated with 5 mg/kg/d PFHxS were used for subsequent tests.

### Effect of PFHxS exposure on the HPG axis

**Fig. 4:**
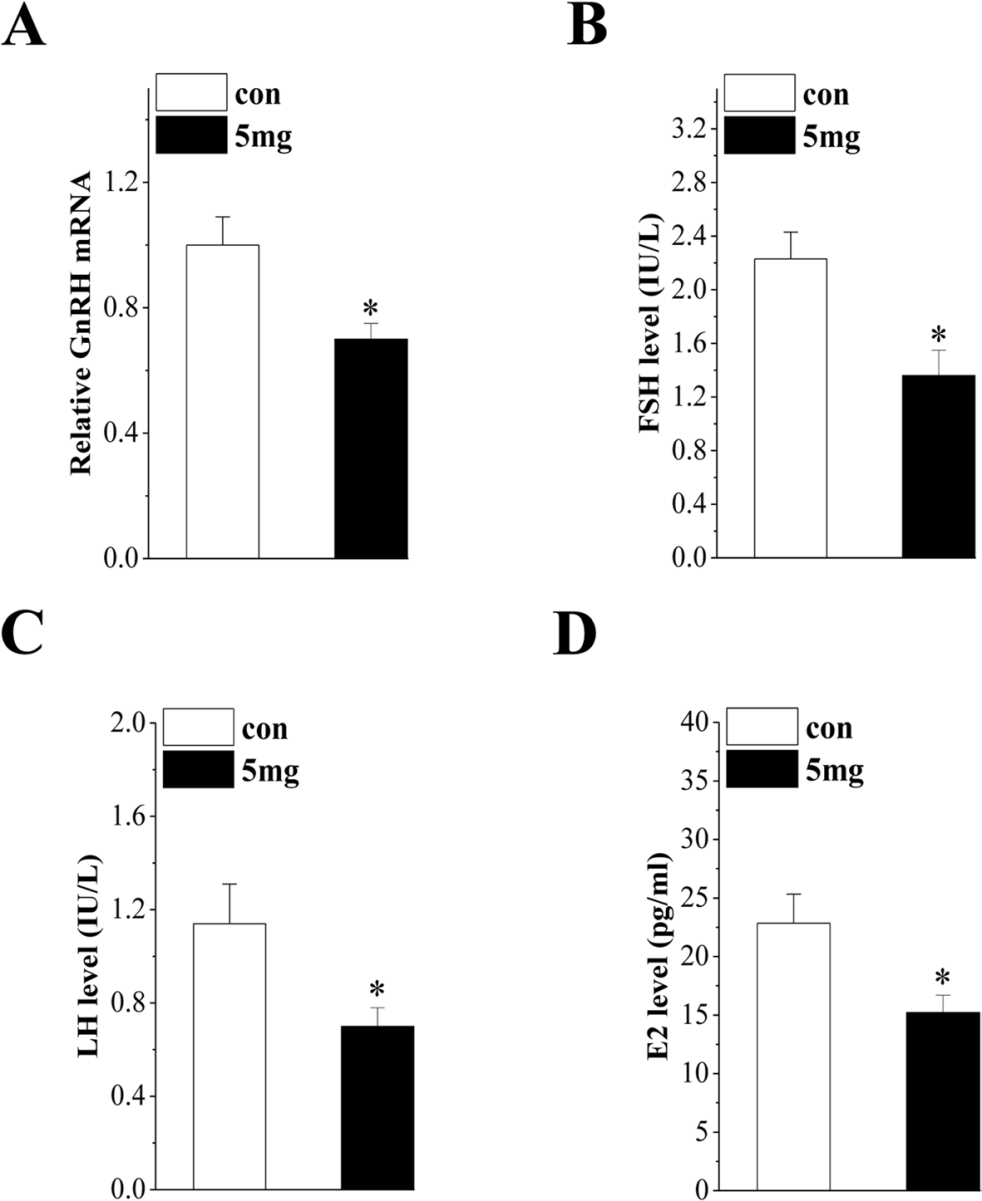
Effect of PFHxS exposure on the HPG axis. Bar graphs show the levels of GnRH mRNA (**A**), serum FSH (**B**), LH (**C**), and E2 (**D**). *P < 0.05 vs. the control mice (*n* = 6/group, Student’s t-test)

**Fig. 5:**
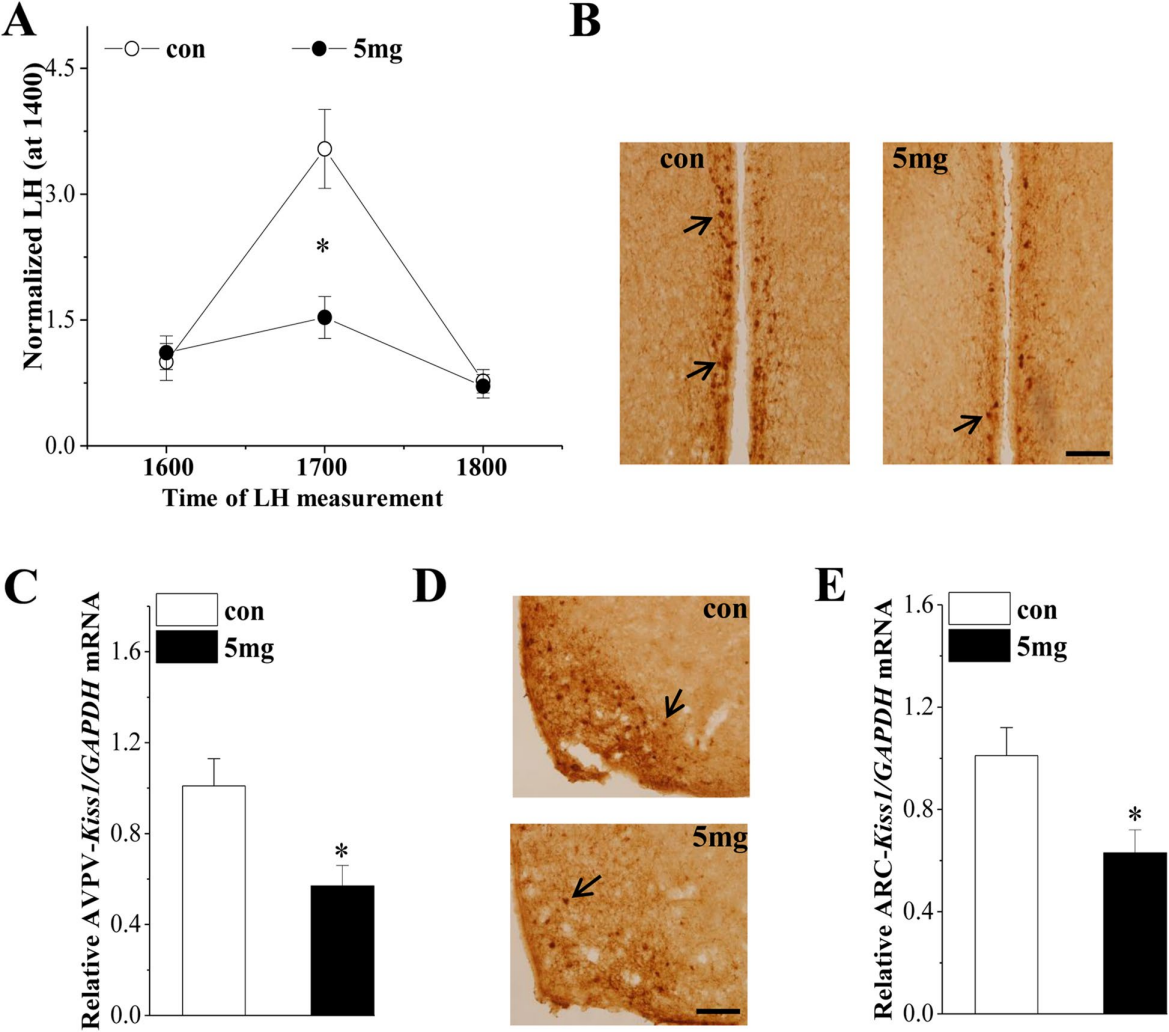
Effect of PFHxS exposure on the LH surge and the expression of kisspeptin. **A** Average serum LH level standardized by 1600 h. *P < 0.05 vs. The control mice (*n* = 6/group, two-way ANOVA). **B** Representative image of kisspeptin immunostaining in AVPV. Arrows indicate AVPV-kisspeptin+ cells. Scale bar = 100 μm. **C** Relative levels of AVPV-kiss1/GAPDH mRNA. **D** Representative image of kisspeptin immunostaining in the ARC. Arrows indicate ARC-kisspeptin+ cells. Scale bar = 100 μm. **E** Relative levels of ARC-kiss1/GAPDH mRNA. *P < 0.05 vs. the control mice (*n* = 6/group, Student’s t-test)

To explore the underlying mechanism of PFHxS exposure on follicle development and ovulation, we measured serum hormone levels to investigate the effects of PFHxS on the HPG axis. As shown in Fig. 4, the GnRH mRNA level in the hypothalamus decreased significantly in the PFHxS-treated mice (*P* = 0.013, *n* = 6; Fig. 4A). Compared with those of the control group at diestrus, the serum levels of FSH (*P* = 0.011, *n* = 6; Fig. 4B), LH (*P* = 0.038, *n* = 6; Fig. 4C), and E2 (*P* = 0.025, *n* = 6; Fig. 4D) were significantly reduced in the PFHxS-treated mice. Moreover, a surge in LH release (LH surge) in proestrus was observed between 1600 and 1800 in the control but not the PFHxS-treated mice (*P* = 0.020, *n* = 6; Fig. 5A). These results indicated that chronic exposure to PFHxS could inhibit the function of the HPG axis.

### Effect of PFHxS exposure on hypothalamic kisspeptin expression

To identify the possible mechanism of the effect of PFHxS on the HPG axis, we measured kisspeptin expression in the AVPV and ARC. The number of AVPV-kisspeptin+ cells (Fig. 5B) and the level of AVPV-kiss-1 mRNA (*P* = 0.015, *n* = 6; Fig. 5C) were significantly reduced at proestrus after PFHxS exposure. Similarly, the number of ARC kisspeptin+ cells (Fig. 5D) and the level of ARC-kiss-1 mRNA (*P* = 0.021, *n* = 6; Fig. 5E) were also significantly decreased at diestrus in the PFHxS-treated mice. These results indicated that PFHxS suppressed the activation of kisspeptin neurons and reduced kiss-1 mRNA expression.

### Kp-10 restored follicle development and the function of the HPG axis

**Fig. 6:**
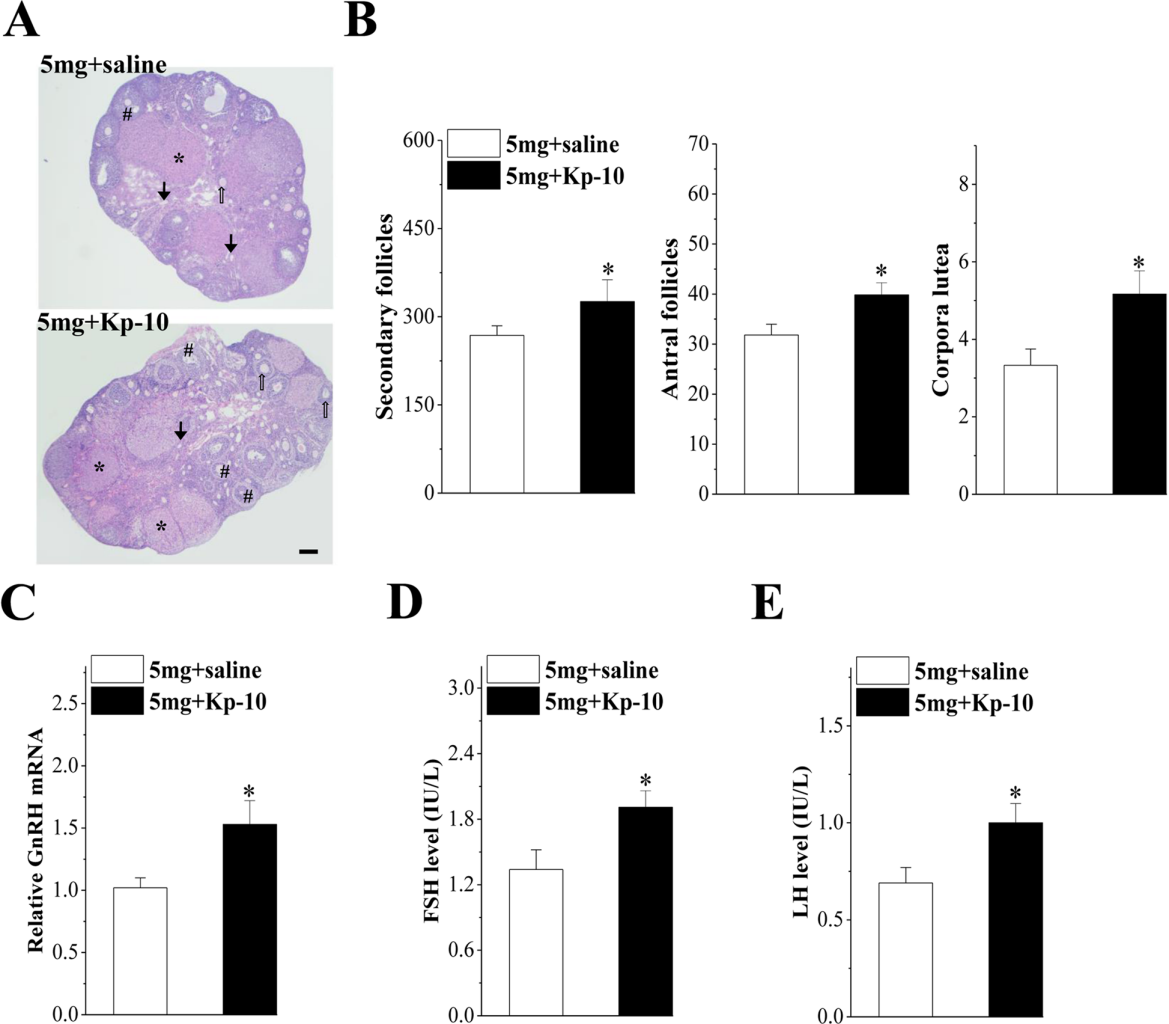
Effect of Kp-10 on follicle development and the function of HPG axis. **A** Representative images of ovaries in the PFHxS+saline and PFHxS+Kp-10 treated mice. * for corpora lutea; # for antral follicles; ⇧ for secondary follicles; ↑ for atretic follicles. Scale bar = 200 μm. **B** Average number of secondary follicles, antral follicles and corpora lutea at diestrus. **C**-**E** Relative levels of GnRH mRNA, serum FSH, and LH levels in the PFHxS+saline- and PFHxS+Kp-10-treated mice. *P < 0.05 vs. The PFHxS+saline-treated mice (*n* = 6/group, Student’s t-test)

As shown in Fig. 6A and B, the numbers of secondary follicles (*P* = 0.039, *n* = 6), antral follicles (*P* = 0.034, *n* = 6) and corpora lutea (*P* = 0.032, *n* = 6) significantly increased after treatment with Kp-10 in the mice given 5 mg/kg/d PFHxS. Furthermore, the GnRH mRNA level (*P* = 0.031, *n* = 6; Fig. 6C), and serum levels of FSH (*P* = 0.033, *n* = 6; Fig. 6D) and LH (*P* = 0.034, *n* = 6; Fig. 6E) were also higher in the PFHxS+Kp-10 mice than in the PFHxS+saline mice. These results indicated that Kp-10 could partially restore follicle development and that the function of the HPG axis was suppressed by PFHxS exposure.

## Discussion

The present study provides in vivo evidence that chronic exposure to PFHxS affects HPG axis functions by inhibiting the expression of kisspeptin in the ARC and AVPV regions. This process disrupts follicular development and ovulation in mice. The toxicity of PFHxS can be overcome by subsequent administration of Kp-10.

The reproductive endocrine system in women is regulated by the HPG axis, which is affected by many factors including genetic, iatrogenic, environmental, psychological, and nutritional factors [30, 31]. Endocrine disruptors (EDCs) are exogenous chemical substances that can mimic natural hormones thereby exerting agonistic or antagonistic effects and interfering with their actions [32]. PFASs are a kind of EDCs that resist degradation, and persist in environmental and biological samples [33, 34]. In our previous study, we analysed the levels of 10 common PFASs in the plasma of 120 patients with premature ovarian insufficiency (POI) and 120 matched controls. We found that the plasma levels of PFOA, PFOS, and PFHxS in the patients were significantly higher than those in the controls, and high exposure to PFOA, PFOS, and PFHxS was associated with an increased risk of POI [35]. Another epidemiological study in Shanghai showed that increased exposure to PFOA, PFOS, and PFHxS was related to menstrual cycle disorders [36]. The underlying molecular mechanisms of PFOA and PFOS toxicity on reproduction have been analysed in many in vivo and in vitro studies in recent years [37, 38], while the effect and possible mechanism of PFHxS on reproduction remain elusive and need to be further explored.

In humans, PFHxS is mainly distributed in plasma proteins, liver, and kidney [39]. The elimination of PFHxS mainly depends primarily on reabsorption and filtration by the proximal tubules of the kidney [40]. A previous study found that menstruation was another pathway for the removal of PFOA and PFOS, and that this process may also be involved in the clearance of PFHxS [41]. Current epidemiological studies on PFHxS have mainly focused on its effects on thyroid, liver, and immune functions [42–44], whereas very few reports have analysed its effect and mechanism on reproductive functions. To investigate the possible mechanism underlying the adverse effect of PFHxS on reproduction, we tried to establish a mouse model of chronic PFHxS exposure. The median and interquartile range of PFHxS was 0.29 (0.22–0.37) ng/mL in our previous study [35] and 0.69 (0.56–0.88) ng/mL reported in another epidemiological study [36] respectively. In human, PFHxS accumulates over the years, which is a low dose and long-term outcome. In animals, it is difficult to produce circulating concentrations of PFHxS that can reflect human exposure. The remaining studies concerning the animal models of PFHxS exposure are very limited. Researchers once administered 0.3, 1.0 and 3.0 mg/kg/d PFHxS to CD-1 mice for at least 42 days to evaluate the reproductive/developmental toxicity in a previous study [45]. Another 42-day model of PFHxS exposure involved doses of 0.3, 1.0, 3.0 and 10 mg/kg/d in Sprague-Dawley rats [11]. Based on these previous studies, mice in the present study were given PFHxS at doses of 0.5, 5, and 50 mg/kg/d by oral gavage for 42 days. When the study finished, we found that PFHxS exposure at a dose of 50 mg/kg/d significantly reduced the mean body and ovarian weights respectively, whereas the relative ovary to body weight in the PFHxS-treated mice was not significantly different from that in the controls. This result might suggest that 50 mg/kg/d PFHxS administration leads to the systemic toxicity in mice but is not especially damaging to the ovary. To avoid the adverse influence of the systemic toxicity on reproductive functions, we analysed only mice treated with 0.5 and 5 mg/kg/d PFHxS for the subsequent experiment.

In comparison with the regular estrous cycle observed in the control mice, the mice treated with 5 mg/kg/d PFHxS had a prolonged estrous cycle, especially an increased duration of diestrus. Moreover, the mice given 5 mg/kg/d PFHxS showed a significant decrease in the numbers of secondary follicles, antral follicles and corpora lutea, suggesting a deficit of follicle development and maturation. As no obvious adverse effect on reproduction was observed in the 0.5 mg/kg/d group, a dose of 5 mg/kg/d was considered appropriate for this mouse model of chronic exposure to PFHxS. The HPG axis regulates reproductive endocrine functions. We found that the serum levels of FSH, LH, and E2 were obviously reduced in the PFHxS-treated mice. Moreover, the GnRH mRNA level in the hypothalamus showed an obvious decrease while the GAPDH Ct values were unchanged after PFHxS exposure (F_(3,20)_ = 1.408, *P* > 0.05, *n* = 6; Supplemental Fig. 1). These results suggest that PFHxS affects the synthesis or release of HPG hormones, and this effect may occur at the hypothalamic level.

Thyroid function is important to maintain female homeostasis and normal reproductive functions. Some prior studies have shown that perinatal exposure to PFHxS induces adverse effects on thyroid function in rats and is capable of lowering serum thyroxine (T4) [2, 46]. A meta-analysis once identified a negative correlation between PFHxS and total T4 in adults, but did not identify an effect on free T4, total T3 or thyroid stimulating hormone (TSH) [47]. In the present study, the serum level of T4 was not significantly different from that of the controls after PFHxS exposure at a dose of 5 mg/kg/d (*P* > 0.05, *n* = 6; Supplemental Fig. 2). It seemed that 5 mg/kg/d PFHxS exposure to mice for 42 days did not induce adverse effects on thyroid function in mice. This result was consistent with our clinical research showing that the levels of plasma PFHxS in neither POI patients nor the controls were associated with the concentration of T3, T4 and TSH.

Generation of the LH peak before ovulation is an important factor in ovulation and a normal estrous cycle [48]. Changes in the pattern (time and amplitude) of the LH peak may directly disrupt ovarian function [49]. After chronic PFHxS exposure, the LH pulse disappeared, which may at least partly explain the follicular development disorder. The kiss-1 gene expresses kisspeptin and may participate in regulating the HPG axis. Extensive evidence indicates that AVPV-kisspeptin neurons regulate the generation of GnRH and the LH surge, and ARC-kisspeptin neurons are involved in the rhythm of the GnRH pulse [50]. Smith et al. analysed kiss-1 mRNA expression in the AVPV and ARC in response to E2 and found that E2 could stimulate AVPV kisspeptin neurons and inhibit ARC kisspeptin neurons [51, 52]. Another study found that central or peripheral administration of kisspeptin had a strong stimulating effect on gonadal hormones [53]. Low dose intraventricular injection of kisspeptin could significantly increase the secretion of LH and FSH [54], which is expressed by activating GnRH neurons. To confirm whether the toxic effect of PFHxS on reproduction was associated with the activity and synthesis of kisspeptin, we detected its expression levels in the AVPV and ARC areas of the hypothalamus. We found that the numbers of kisspeptin-positive neurons in the AVPV and ARC areas decreased simultaneously, along with the mRNA level of kiss-1 in the hypothalamus.

To verify the role of kisspeptin in PFHxS-mediated toxicity, we injected Kp-10 into the lateral ventricle of the mice and found that the reproductive toxicity caused by PFHxS improved to a certain extent. Specifically, a significant increase in the numbers of secondary follicles, antral follicles and corpus lutea was observed, along with an increase in gonadal hormone levels. This result further supports our hypothesis that PFHxS may affect the HPG axis by reducing the expression of kisspeptin in the hypothalamus, thereby affecting follicular development and ovulation in mice.

Few studies have reported mild to no effects of PFHxS on reproduction in mice. One such study by Chang et al. found that daily PFHxS treatment did not affect the weight, food consumption, hematology, serum TSH level, or reproductive function in mice, including the estrous cycle, fertility, conception, pregnancy, and birth [45]. However, that study used a maximum PFHxS dose of 3 mg/kg/d, whereas we studied the effect of 5 mg/kg/d PFHxS. This difference in dose may be a factor in the differing results we obtained.

To date, several epidemiological studies have identified associations between PFHxS and altered liver, thyroid, energy and lipid metabolism, protein biosynthesis, and immune function, which may be indirectly involved in the reproductive toxicity caused by PFHxS [55–58]. In addition, AVPV- and ARC- kisspeptin neurons express prolactin receptors, suggesting the possible participation of prolactin in the toxicity of PFHxS [59, 60]. It is still unclear whether PFHxS can suppress the biosynthesis of E2 directly, similar to PFOS, by reducing histone acetylation of the steroidogenic acute regulatory protein (StAR) [61]. All of the above issues are problems waiting to be solved and we would like to address these problems in our future studies.

## Conclusions

Although the mechanism underlying PFHxS-induced suppression of kisspeptin expression remains elusive, our data in the present study indicate that chronic exposure of mice to PFHxS at a dose of 5 mg/kg/day affects reproductive function by inhibiting kisspeptin expression in the ARC and AVPV regions, leading to deficits in follicular development and ovulation. These findings may be helpful for understanding the effect of PFHxS exposure on reproductive endocrine functions and reproductive health in humans.

## Supplementary Information

**Additional file 1: S-Fig. 1.** Effect of PFHxS exposure on reference genes (GAPDH) for the RT-qPCR. Bar graphs show levels of cycle threshold (Ct) values. (*n* = 6/group, one-way ANOVA). **S-Fig. 2.** Levels of T4 in the control and PFHxS mice. Bar graphs show levels of T4. (*n* = 6/group, Student’s t-test).

## Data Availability

All the data is contained in the manuscript.

## Abbreviations

PFHxS: Perfluorohexane sulfonate
GnRH: Gonadotropin releasing hormone
FSH: Follicle-stimulating hormone
LH: Luteinizing hormone
E2: Estrogen
POA: Preoptic area
ARC: Arcuate nucleus
AVPV: Anteroventral periventricular nucleus
Kp-10: Kisspeptin-10
PFASs: Perfluoroalkyl compounds
EDCs: Endocrine-disrupting chemicals
DMSO: Dimethyl sulfoxide
GPR54: G proteincoupled receptor 54
DAB: Diamino benzidine
DNA: Deoxyribonucleic acid
ECL: Enhanced chemiluminescence
ELISA: Enzyme-linked immuno sorbent assay
H&E: Hematoxylin and eosin
