# Supplementary material for: Chronic exposure to perfluorohexane sulfonate leads to a reproduction deficit by suppressing hypothalamic kisspeptin expression in mice
Source: J Ovarian Res. 2021 Oct 27;14:141. doi: 10.1186/s13048-021-00903-z (PMC8555149; doi:10.1186/s13048-021-00903-z)

**Supplementary materials**

Manuscript: JOVR-D-21-00235

Chronic exposure to perfluorohexane sulfonate leads to a reproduction deficit by suppressing hypothalamic kisspeptin expression in mice

**Xiaorui Yin, Tingting Di, Xinyuan Cao, Zhengnan Liu, Jingyan Xie, Suyun Zhang**

| Page 1 | S-Figure 1: Cycle threshold values of GAPDH in each group |
| --- | --- |
| Page 2 | S-Figure 2: Levels of T4 in the control and PFHxS mice |
| Page 3 | The certification provided by the Nature Research Editing Service |

Supplemental Figure 1 (S-Fig. 1)


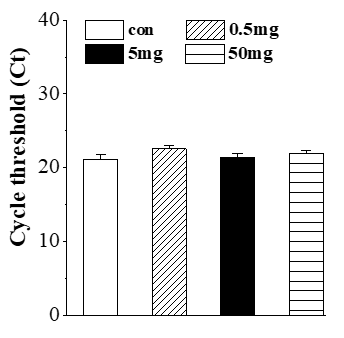


**S-Fig. 1.** Effect of PFHxS exposure on reference genes (GAPDH) for the RT-qPCR. Bar graphs show levels of cycle threshold (Ct) values. (n=6/group, one-way ANOVA)

Supplemental Figure 2 (S-Fig. 2)


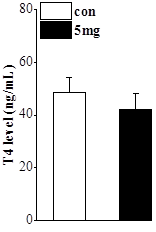


**S-Fig. 2.** Levels of T4 in the control and PFHxS mice. Bar graphs show levels of T4. (n=6/group, Student's t-test)


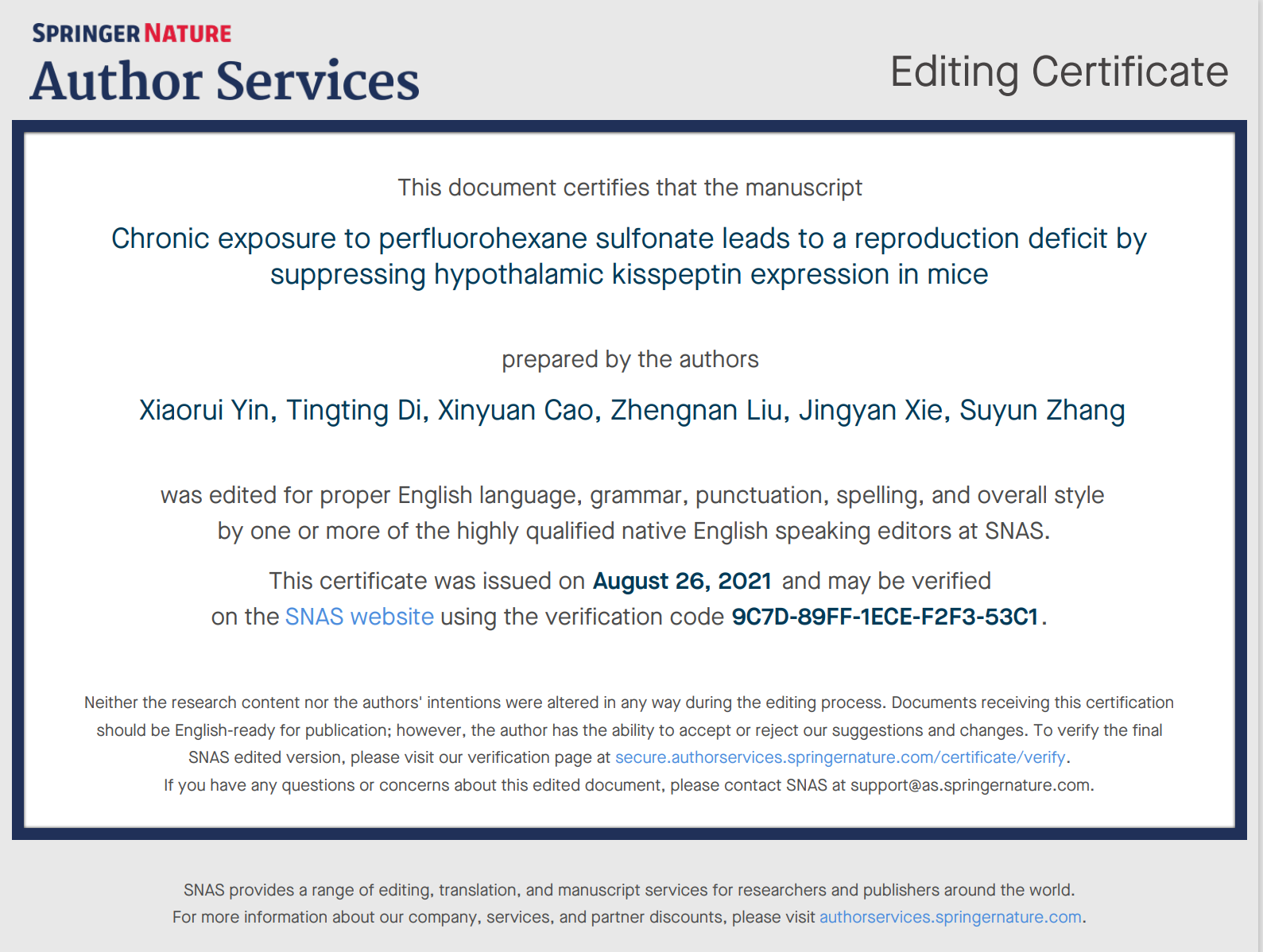

Supplement: Supplementary file 1 — Additional file 1: S-Fig. 1. Effect of PFHxS exposure on reference genes (GAPDH) for the RT-qPCR. Bar graphs show levels of cycle threshold (Ct) values. (n = 6/group, one-way ANOVA). S-Fig. 2. Levels of T4 in the control and PFHxS mice. Bar graphs show levels of T4. (n = 6/group, Student’s t-test). [file 13048_2021_903_MOESM1_ESM.doc]
